# Human Crimean-Congo Hemorrhagic Fever, Sénégal

**DOI:** 10.3201/eid1010.040586

**Published:** 2004-10

**Authors:** Pierre Nabeth, Moussa Thior, Ousmane Faye, François Simon

**Affiliations:** * Institut Pasteur de Dakar, Dakar, Sénégal;; † Centre de santé, Popenguine, Sénégal

**Keywords:** letter, Crimean-Congo, hemorrhagic fever, Sénégal, human, serology

**To the Editor:** Crimean-Congo hemorrhagic fever (CCHF) virus, genus *Nairovirus*, family *Bunyviridae*, is transmitted to mammals and birds by ticks. *Hyalomma* ticks, the primary vectors in CCHF transmission, are widespread throughout Europe, Asia, the Middle East, and Africa; evidence of CCHF virus has been found in all these regions. CCHF in humans is an acute viral disease that is transmitted by the bite of infected ticks, direct contact with blood or infected tissues from viremic animals, and direct contact with the blood or secretions of an infected person (*1*).

On January 26, 2003, a 22-year-old shepherd was treated at a health post in the Popenguine District, 60 km south of Dakar, Sénégal; he reported fever, epistaxis, arthralgia, myalgia of the lower limbs, and dark urine for the past 2 days. Without biologic confirmation of the infection, he was treated for malaria with two intravenous injections of quinine, followed by oral administration of chloroquine.

On January 31, the patient had a temperature of 39°C, conjunctival jaundice, bleeding gums, and was vomiting blood. He was seen again at the health post and was given antimicrobial drugs, intravenous quinine, and vitamin K; the next day, the bleeding stopped and the fever subsided. A serum sample was sent to the World Health Organization Collaborative Centre for Arboviruses and Viral Hemorrhagic Fevers at the Institut Pasteur, Dakar. Tests for anti-CCHF specific immunoglobulin (Ig) M antibody by enzyme-linked immunosorbent assay (ELISA) were positive, and CCHF virus by isolation on cell cultures (AP61 and Vero cells) and reverse transcriptase-polymerase chain reaction (RT-PCR) were negative. From January 31 to February 10, the IgM titer increased from 1/3,200 to >1/12,800 and IgG titer increased from 1/200 to 1/6,400.

Examination of the patient on February 10 showed he had recovered without sequelae, and no trace of tick bites was found. The patient stated that he had not traveled, noticed any tick bites, slaughtered any animals, or been in contact with people with fever for several weeks before his illness. He lived in close proximity to goats and cattle, but no blood samples were taken from these animals. Although no ticks were found on nearby goats, 10 *Amblyomma* and *Hyalomma* ticks were collected from three cattle. Ticks were negative for CCHF virus isolation on suckling mice and RT-PCR amplification.

No other case of fever accompanied by hemorrhage was reported in the area, and none of the patient's 14 close contacts became ill. Of the four close contacts from whom blood samples were taken, analyses for IgM and IgG antibodies against CCHF virus were negative by ELISA.

While no clinical case of CCHF has ever been reported in Senegal, studies dating from 1969 indicate that CCHF virus had been found in various locations in the country (*2**,**3*). In the village of Bandia, in the same district where the reported case was observed, a study conducted from 1986 to 1988 showed a prevalence of anti-CCHF IgG of 3.2% in the human population (*4*). Another study, conducted in the same area from 1989 to 1992, showed seroconversions for several ruminants and isolated the virus from ticks (*5*).

During CCHF outbreaks, an average of 30% of people who had the disease died (case-fatality ratio). It is often discovered during nosocomial outbreaks, as was the case in Mauritania, a country on Sénégal's northern border, in 2003 (P. Nabeth, unpub. data). To prevent outbreaks of CCHF, public awareness campaigns aimed at the populations most at risk—livestock farmers, butchers, and health personnel—must be conducted, and the epidemiologic alert systems must be strengthened. In addition, conditions that enhance maintenance of the virus in nature and its transmission to humans must be better understood so adequate control measures can be developed.
